# Systemic lupus erythematosus combined with Wilson’s disease: a case report and literature review

**DOI:** 10.1186/s12887-024-04713-2

**Published:** 2024-04-15

**Authors:** Zhenle Yang, Qian Li, Suwen Liu, Zihan Zong, Lichun Yu, Shuzhen Sun

**Affiliations:** 1 Department of Pediatric Nephrology and Rheumatism and Immunology, Cheeloo College of Medicine, Shandong Provincial Hospital, Shandong University, Jinan, 250021 P. R. China; 2 Department of Pediatric Nephrology and Rheumatism and Immunology, Shandong Provincial Hospital Affiliated to Shandong First Medical University, Jinan, 250021 P. R. China

**Keywords:** Systemic lupus erythematosus, Wilson’s disease, Diagnosis, Treatment

## Abstract

**Background:**

Systemic lupus erythematosus (SLE) and Wilson’s disease (WD) are both systemic diseases that can affect multiple organs in the body. The coexistence of SLE and WD is rarely encountered in clinical practice, making it challenging to diagnose.

**Case report:**

We present the case of a 9-year-old girl who initially presented with proteinuria, haematuria, pancytopenia, hypocomplementemia, and positivity for multiple autoantibodies. She was diagnosed with SLE, and her blood biochemistry showed elevated liver enzymes at the time of diagnosis. Despite effective control of her symptoms, her liver enzymes remained elevated during regular follow-up. Laboratory tests revealed decreased serum copper and ceruloplasmin levels, along with elevated urinary copper. Liver biopsy revealed chronic active hepatitis, moderate inflammation, moderate-severe fibrosis, and a trend towards local cirrhosis. Genetic sequencing revealed compound heterozygous mutations in the ATP7B gene, confirming the diagnosis of SLE with WD. The girl received treatment with a high-zinc/low-copper diet, but her liver function did not improve. Upon recommendation following multidisciplinary consultation, she underwent liver transplantation. Unfortunately, she passed away on the fourth day after the surgery.

**Conclusions:**

SLE and WD are diseases that involve multiple systems and organs in the body, and SLE complicated with WD is rarely encountered in the clinic; therefore, it is easy to misdiagnose. Because penicillamine can induce lupus, it is not recommended. Liver transplantation is indicated for patients with liver disease who do not respond to medical treatment with WD. However, further research is needed to determine the optimal timing of liver transplantation for patients with SLE complicated with WD.

## Background

Wilson’s disease (WD) is a rare autosomal-recessive disorder characterized by defective biliary excretion of copper, with a reported prevalence of 1:30,000–50,000 [1]. This defect leads to the progressive accumulation of copper in various organs and tissues, particularly the liver, corneas, kidneys, heart, and nervous system. Systemic lupus erythematosus (SLE), a multisystem autoimmune inflammatory disease, can affect any organ in the body. However, there is limited literature focusing on the coexistence of WD and SLE. In this case report, we report the occurrence of SLE and WD in a 9-year-old girl to improve the understanding of the coexistence of these two conditions.

## Case report

In October 2019, a 9-year-old girl presented to our centre with a complaint of red-coloured urine lasting for 1 week. She had no accompanying symptoms, such as rash, jaundice, oliguria, joint swelling, fever, or neurological symptoms, or a significant family history. Urinalysis revealed the presence of more than 80% dysmorphic red blood cells along with proteinuria but no crystalluria or bacterial growth. Her total leucocyte count was 2.02 × 10^9/L, her haemoglobin level was 10.5 g/dl, her platelet count was 105 × 10^9/L (within the range of 150–300), her aspartate aminotransferase (AST) level was 116 U/L (normal range: 10–40), her alanine aminotransferase (ALT) level was 145 U/L (normal range: 10–40), her serum alkaline phosphatase level was 334 U/L (normal range: 140–560), her blood urea level was 5.2 mmol/L (normal range: 2.8–7.4), her serum creatinine level was 48.0 µmol/L (normal range: 40–80), her serum albumin level was 25.7 g/L (normal range: 40–55), her cholesterol level was 3.02 mmol/L (normal range: 3.6–6.2), her serum bilirubin level was 4.98 µµmol/L (normal range: 3.5–23.5), and her complement C3 level was 0.2 g/L (normal range: 0.8–1.8). Anti-nuclear antibody (ANA) and anti-double-stranded DNA antibody (anti-dsDNA) tests were positive. Abdominal ultrasound revealed no abnormalities. A kidney biopsy was performed to determine the renal pathology. We detected IgA(+), IgG(+), C3(+), F+(−), IgM(++), and C1q(++) by immunofluorescence. Under light microscopy, we detected two cases of focal segmental sclerosis with balloon adhesions and one case of cell-fibrous neoplasm formation in 26 glomeruli. Diffuse light-moderate proliferation was present in the mesangial zone. There was no thickening of the glomerular basement membrane (GBM). Podocyte swelling, swelling of epithelial cells and focal fibrosis of the renal interstitium were observed. The patient was diagnosed with SLE and lupus nephritis (LN). She was regularly followed up at the outpatient department, and her symptoms were effectively controlled with methylprednisolone, hydroxychloroquine (HCQ), and mycophenolate mofetil (MMF), except for her persistently elevated liver enzymes.

**Fig. 1:**
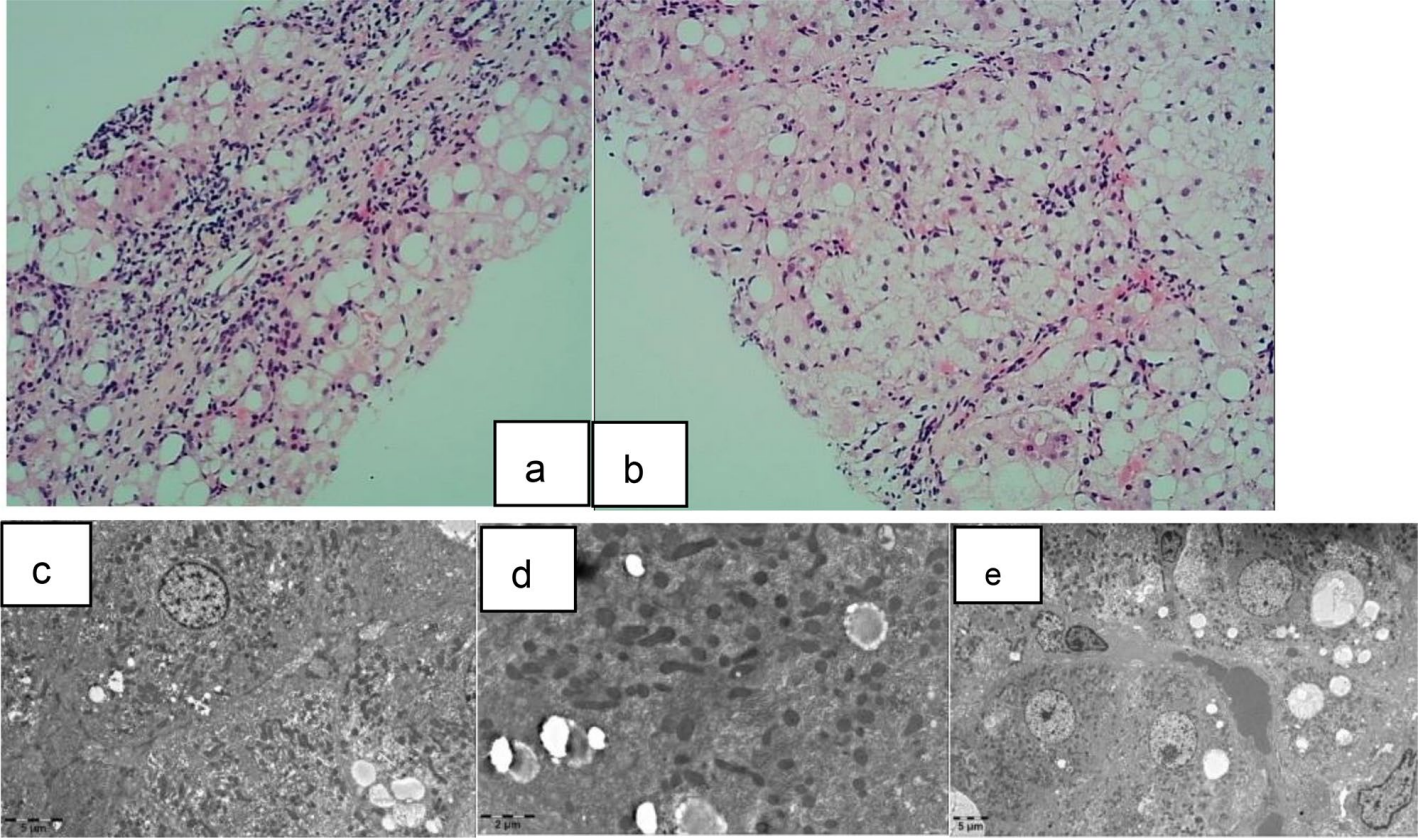
Liver biopsy sample from the patient. a and b: Inflammation and interface hepatitis in the portal area, hepatocyte steatosis, and bridging fibrosis. c, d, and e: Hepatocyte swelling, lipid droplets of varying sizes within some hepatocytes, the presence of cholesterol crystals, and dense collagen fibre deposition in the portal area accompanied by lymphocyte and plasma cell infiltration

**Fig. 2:**
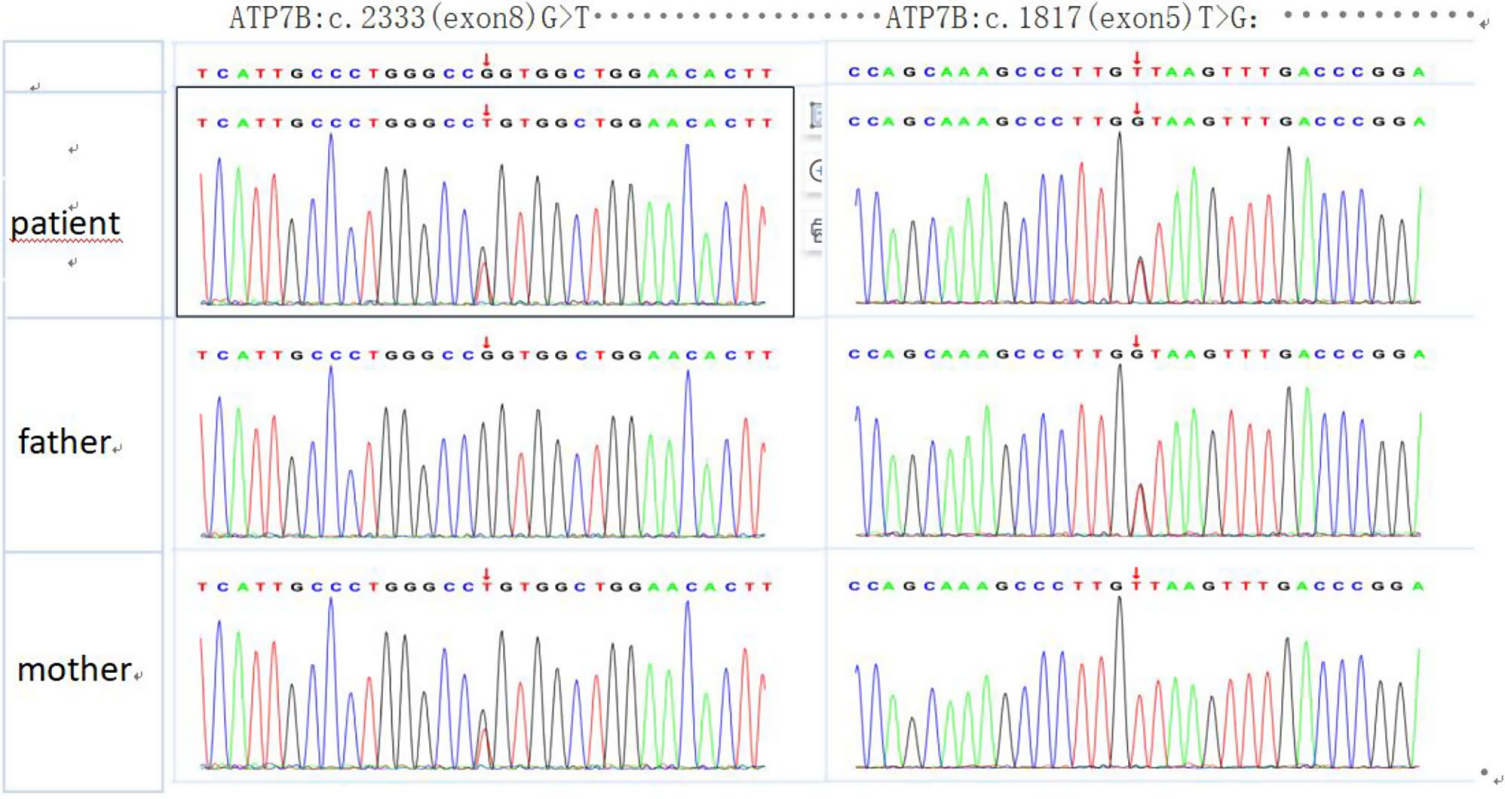
Sequence analysis of ATP7B in the family. The patient had compound heterozygous mutations in ATP7B: the maternally inherited mutation was c.2333(exon8)G > T, p.R788L, while the paternally inherited mutation was c.1817(exon5)T > G, p.V606G

During her routine annual health checkup conducted in February 2022, her serum biochemistry still showed abnormalities in hepatic function: AST, 105 U/L; ALT, 61 U/L; and GGT, 138 U/L. However, the results of urine analysis, complete blood count, renal function tests, multiple autoantibody tests, and complement component analysis were within normal limits. The cause of the abnormal hepatic function remained unexplained, as her SLE Disease Activity Index (SLEDAI) score was 0. She exhibited no signs of abdominal distension, anorexia, or yellow staining of the skin or mucous membranes. She denied using any medications or substances that could have detrimental effects on the liver, and she had no history of infectious disease or neurological disturbances. Physical examination revealed no abnormalities in the abdomen or nervous system. Additionally, the patient tested negative for a range of autoimmune liver disease antibodies, hepatitis viruses, Epstein‒Barr virus, cytomegalovirus, and tumour markers during the follow-up. To investigate whether abnormal hepatic function was induced by medication, we advised her parents to discontinue the use of HCQ and MMF. However, the total leucocyte count decreased to 2.06 × 109/L, the complement C3 level decreased to 0.63 g/L, and the anti-dsDNA antibody level increased to 178.61 IU/ml (0-100), owing to which MMF and HCQ were discontinued after three weeks. Serum biochemistry still revealed elevated liver enzymes: AST level: 144 U/L; ALT level: 105 U/L; and GGT level: 159 U/L. Serum ceruloplasmin and tumour marker tests were also conducted. Interestingly, there was a significant decrease in serum ceruloplasmin (< 9.5 mg/dl; normal range 20–60 mg/dl), but there were no Kayser–Fleischer (K-F) rings in either eye. Therefore, serum copper and urinary copper were measured, and laboratory tests revealed that the serum copper level decreased (1.45 µmol/L; normal range 12.6–23.6 µmol/L), while the urinary copper level increased (213 µg/24 h; normal range 15–30 µg/24 h). MRI was performed to evaluate the patient’s liver. Liver MRI revealed that the liver was plump, and multiple nodules with long T1 and short T2 abnormal signals were observed in the liver parenchyma. Liver biopsy and gene sequencing were subsequently conducted. Liver biopsy sample analysis demonstrated a trend towards chronic active hepatitis, moderate inflammation, moderate-severe fibrosis, and local cirrhosis (Fig. 1). The liver was negative for copper content. Sequencing (Fig. 2) revealed that the patient (proband) had compound heterozygous mutations in ATP7B. The maternally inherited mutation (c.2333(exon8)G > T, p.R788L) was previously reported to be pathogenic, and the paternally inherited mutation (c.1817(exon5)T > G, p.V606G) was likely pathogenic according to the American College of Medical Genetics (ACMG) guidelines (2019). Consequently, a final diagnosis of SLE with WD was made. Due to the potential induction of lupus by penicillamine use, the patient was treated with a high-zinc/low-copper diet, and the amount of methylprednisolone was increased because of the reactivation of her SLE. The total leucocyte count decreased to 6.73 × 109/L, the complement C3 level increased to 0.85 g/L, and the anti-dsDNA antibody level decreased to 145.32 IU/ml (0-100); unfortunately, her liver function did not improve. Upon recommendation following multidisciplinary consultation, the patient underwent liver transplantation; however, she tragically passed away on the fourth day after surgery despite having a good clinical situation before surgery.

## Discussion

SLE is a chronic, systemic autoimmune disease that primarily affects young women. SLE is characterized by a complex and heterogeneous clinical presentation, with the potential to involve multiple organs and tissues throughout the body. The liver is an important target organ in SLE. Reports indicate that 25-50% of SLE patients may experience liver abnormalities during the course of the disease [1]. Although the role of SLE in the development of asymptomatic liver disease is still debated, many experts recognize that SLE often leads to subclinical liver dysfunction, known as lupus hepatitis [2]. Lupus hepatitis is a nonspecific reactive liver disease primarily caused by complement deposition and vasculitis-induced organic damage in the liver [3, 4]. Lupus hepatitis is frequently associated with SLE flares or clinical activity, and it can be diagnosed only by ruling out secondary causes of liver involvement [2]. In the reported case, the patient was diagnosed with SLE and presented with asymptomatic elevation of liver enzymes. We ruled out drug-induced liver injury, fatty liver disease, autoimmune liver disease, and viral hepatitis, suggesting that the asymptomatic increase in liver enzymes was likely due to SLE itself. However, despite active treatment for SLE and improvements in other symptoms, the asymptomatic increase in liver enzymes persisted for more than 2 years, which prompted us to actively search for the underlying cause.

Previous studies have indicated that drug-induced liver injury is a major cause of abnormal liver function in SLE patients, with an occurrence rate of approximately 31% in SLE patients with liver dysfunction [5]. SLE patients are prone to drug-induced liver injury due to their high levels of oxidative stress [6]. Nonsteroidal anti-inflammatory drugs (NSAIDs), azathioprine, and methotrexate are the most common drugs associated with liver injury, followed by cyclophosphamide and leflunomide. HCQ, MMF, cyclosporine, tacrolimus, and corticosteroids rarely cause liver injury. In most cases, drug-induced liver dysfunction is mild and transient, and liver function can usually recover with a dose reduction or discontinuation of the medication [7, 8]. Although HCQ and MMF rarely cause liver damage, we recommended discontinuing these medications to prevent drug-induced liver injury. However, after the discontinuation of these medications, the patient’s liver function did not improve. Instead, the SLE itself became active.

Autoimmune hepatitis (AIH) is a chronic liver disease of unknown aetiology characterized by a T-cell-mediated immune response against liver self-antigens, leading to hepatocyte necrosis and inflammation. Diagnosis is based on elevated serum transaminases, increased levels of IgG, the presence of autoantibodies (ANA, anti-smooth muscle antibodies, and anti-liver-kidney microsome type 1 antibodies), and histological findings in liver biopsy samples, such as interface hepatitis and lymphoplasmacytic infiltrates [9]. The occurrence rate of AIH in SLE patients with liver dysfunction is approximately 5-10% [10, 11]. In the patient whose case is reported here, AIH-related antibodies were negative, but the liver biopsy sample showed pathological features similar to those of AIH, although they were nonspecific.

SLE patients receiving immunosuppressive therapy are more susceptible to viral infections, which can result in liver dysfunction [10]. The patient was screened for hepatitis B virus, hepatitis C virus, Epstein‒Barr virus, herpes simplex virus, varicella-zoster virus, and human immunodeficiency virus, and the results of all these tests were negative.

WD is a rare autosomal recessive disorder characterized by copper metabolism dysfunction due to mutations in the ATP7B gene. The resulting copper toxicity primarily affects the liver and brain [12]. WD is classified into different types based on symptoms, including hepatic, neurological, mixed, and other types. In adolescents, the hepatic type has a greater occurrence rate than the other types. The liver manifestations in WD patients can vary, ranging from asymptomatic elevation of liver enzymes to significant liver cirrhosis (compensated or decompensated) or acute liver failure. Reports indicate that 18-23% of WD patients experience asymptomatic elevation of liver enzymes [13]. Similarly, the most common liver manifestation in SLE patients is asymptomatic elevation of liver enzymes, which was the main reason for the delayed diagnosis of our patient. However, in SLE patients who respond well to effective treatment, liver function abnormalities typically improve. Therefore, in SLE patients with well-controlled disease who present with unexplained elevation of liver enzymes, the possibility of WD should be considered.

K-F rings are a typical ophthalmic manifestation of WD. Reports indicate that K-F rings are present in nearly 100% of patients with neurological WD, 40-50% of patients with hepatic involvement, and 20-30% of asymptomatic patients [14]. In our patient, there were no K‒F rings in either eye. Therefore, the absence of K-F rings does not exclude the diagnosis of hepatic WD. Serum ceruloplasmin measurement is a safe and simple screening test for WD. However, importantly, serum ceruloplasmin can also be decreased in diseases such as Menkes disease, nephrotic syndrome, protein-losing enteropathy, and various chronic liver diseases. Additionally, in 5-15% of WD patients, ceruloplasmin levels may be normal or only slightly lower than the normal range [15]. In the patients with WD treated at our centre, decreased ceruloplasmin levels were detected at the time of diagnosis. However, the presence of LN in the patient who presented with nephrotic syndrome led us to consider that the decreased ceruloplasmin levels may be related to significant protein loss due to LN, which was also a factor contributing to the delayed diagnosis. The measurement of 24-hour urinary copper excretion may be the best screening test for WD, and a value exceeding 100 µg in a 24-hour urine collection has diagnostic value for WD [16]. Therefore, in patients suspected of having hepatolenticular degeneration, serum ceruloplasmin and 24-hour urinary copper excretion should be examined. Especially in patients with significant proteinuria and decreased ceruloplasmin levels where the cause of the decrease cannot be distinguished due to protein loss, further clarification can be achieved through 24-hour urinary copper excretion.

Autoimmune antibodies and autoimmune disorders have been reported in patients with WD. These include ANAs, antiphospholipid antibodies and, rarely, even anti-double-stranded DNA [17, 18]. Antczak-Kowalska M et al. reported that the presence of all studied autoantibodies was greater in WD patients than in healthy individuals [19]. The role of autoantibodies in the pathogenesis of WD is unclear but may be related to a bystander phenomenon. Several autoimmune disorders have been reported in patients with WD, including SLE, myasthenia gravis and inflammatory bowel disease. Penicillamine use is associated with an increased risk of autoimmune diseases. In our study, the patient had no history of oral penicillamine use.

The treatment of WD includes drug therapy, symptomatic therapy, diet therapy, and liver transplantation. Penicillamine is commonly used as the first-line treatment for acute and/or symptomatic WD [12]. However, penicillamine is not recommended for patients with coexisting WD and SLE due to the potential for the induction of lupus. Because WD was not induced by penicillamine use in our patient, we reviewed other reports of WD with SLE not induced by penicillamine use and identified ten other patients in addition to our patient [17, 20–28]. Among these patients, six received therapy with intravenous sodium dimercaptopropane sulfonate and/or oral zinc sulfate, which led to an improvement in WD symptoms [20, 23–27]. In one patient, penicillamine was initially prescribed but was changed to oral zinc due to worsening central nervous system symptoms after two weeks; however, the patient unfortunately passed away 41 days after admission [17]. Plasma exchange was used to treat fulminant liver failure in another patient, but the patient died five days after admission [21]. One patient was treated with penicillamine for 5 years, and the treatment was changed to tetraethylene teramine dihydrochloride. Liver transplantation was performed due to liver fibrosis. The patient is currently free of SLE and has normal liver enzyme levels according to the report [28]. Our patient underwent liver transplantation three months after being diagnosed with WD because her liver enzymes remained abnormal despite receiving oral zinc. Liver transplantation is a curative option because it replaces the affected liver, provides normal ATP7B protein, restores normal biliary copper excretion (preventing disease recurrence), and facilitates the removal of copper from extrahepatic sites where it may be toxic [29]. Liver transplantation is indicated for patients with liver disease who do not respond to medical treatment, who have fulminant or advanced liver failure, and/or who have significant portal hypertension [30]. With advancements in liver transplantation technology, the clinical indications for WD patients receiving liver transplantation have expanded, leading to satisfactory outcomes. Studies in which large databases were analysed have reported one-year and five-year survival rates of approximately 90% in WD children who underwent liver transplantation [31, 32]. There are very few cases of liver transplantation in patients with SLE.

described worldwide. Barthel et al. first reported the use of liver transplantation in three patients with SLE; two did very well, and one experienced hyperacute rejection [28]. Lian EC et al. and F Zazzetti et al. each reported a patient with SLE; both patients underwent transplantation because of liver failure, did not have SLE reactivation and had good long-term survival [33, 34]. These reports show that patients with SLE are capable of undergoing liver transplantation without experiencing SLE reactivation and have good long-term survival. Our patient died four days after liver transplantation. More cases of liver transplantation in patients with SLE are necessary to determine the optimal timing of liver transplantation for patients with SLE, including patients who have SLE complicated by WD.

## Conclusion

SLE and WD are both systemic diseases that can affect multiple organs in the body. The coexistence of SLE and WD is rarely encountered in clinical practice, which can lead to challenges in its diagnosis. Importantly, penicillamine, a commonly used treatment for WD, is not recommended for patients with coexisting SLE due to its potential to induce lupus. Liver transplantation is considered for patients with WD who do not respond to medical treatment. However, further research is needed to determine the optimal timing of liver transplantation for patients with SLE complicated by WD.

## Data Availability

Data is provided within the manuscript.
